# The Economic Utility of Clinical Psychology in the Multidisciplinary Management of Pain

**DOI:** 10.3389/fpsyg.2017.01860

**Published:** 2017-10-31

**Authors:** Emanuele M. Giusti, Giada Pietrabissa, Gian Mauro Manzoni, Roberto Cattivelli, Enrico Molinari, Hester R. Trompetter, Karlein M. G. Schreurs, Gianluca Castelnuovo

**Affiliations:** ^1^Department of Psychology, Catholic University of the Sacred Heart, Milan, Italy; ^2^Istituto Auxologico Italiano IRCCS, Psychology Research Laboratory, Ospedale San Giuseppe, Verbania, Italy; ^3^Faculty of Psychology, eCampus University, Novedrate, Italy; ^4^Department of Psychology, Health and Technology, Centre for eHealth and Wellbeing Research, University of Twente, Enschede, Netherlands

**Keywords:** pain management, clinical psychology, cost-effectiveness, psychometrics, health psychology, clinical health psychology, clinical health interventions, psychotherapy

## Introduction

Chronic musculoskeletal pain is the leading sources of disability worldwide, imposing an enormous burden to both societies and healthcare systems (Vos et al., 2012). Direct medical expenses and indirect costs due to losses in work productivity exceed $200 billion in the US (Ma et al., 2014; Park et al., 2016) and are a major source of concern in Europe (Breivik et al., 2013). Mean *per capita* costs vary from country to country (see Table 1), but are estimated to double the expenses for the care of matched controls (Gore et al., 2012; Hong et al., 2013). Notably, their impact is directly linked both to the severity of the condition and to the presence of mental comorbidities, and can be inflated by concomitant opioid abuse (Baumeister et al., 2012; Manchikanti et al., 2013; Stockbridge et al., 2015; Rayner et al., 2016).

**Table 1:** Direct and indirect annual cost per capita of musculoskeletal conditions.

**Pain condition**	**References**	**Country**	**Type of cost**	**Cost per patient per year**
Low back pain	Pasquale et al., 2014	US	Direct	$3,607
	Gore et al., 2012	US	Direct	$8,386
	Gustavsson et al., 2012	Sweden	Direct and indirect	$9,781
	Becker et al., 2010	Germany	Direct and indirect	€3,579
	Hong et al., 2013	UK	Direct	£1,074
Osteoarthritis	Pasquale et al., 2014	US	Direct	$5,344
	Xie et al., 2016	Various countries	Direct	From $1,442 to $21,335
			Indirect	From $238 to $29,935
	Gustavsson et al., 2012	Sweden	Direct and indirect	$77,98
Rheumatoid arthritis	Pasquale et al., 2014	US	Direct	$4,036
	Boonen and Severens, 2011	Various countries	Direct and indirect	€10,479
	Lundkvist et al., 2008	Various countries	Direct and indirect	From €2,825 to €24,688
Fibromyalgia	Rivera et al., 2009	Spain	Direct and indirect	€9,982
	Knight et al., 2013	US, France, Germany	Direct and indirect	From $9,199 to $13,518
	Pasquale et al., 2014	US	Direct	$1,755

In the last decades, the biopsychosocial model has attempted to answer to the growing imperative need to identify the best practices for the prevention and treatment of chronic pain and related conditions. Scientific research shows that clinical psychology plays a key role within the multidisciplinary approach that is increasingly being suggested for pain management. Its added value is revealed not only by the improvement of the patient experience, but also with regards to economic savings and cost reduction of his care, which is an issue on which modern health services base their strategic decisions. These benefits have been corroborated by studies addressing psychological treatments for chronic musculoskeletal pain, which will be discussed later. However, we argue that the work of clinical psychologists can improve the economic sustainability of chronic pain management in all the stages of the care, from the assessment phase to the rehabilitation period, providing a differentiated contribution depending on the treatment course of the patient (i.e. conservative treatment, surgical intervention). In particular, we suggest that the cost-effectiveness of chronic pain management can be enhanced employing a psychometrically sound, computerized and integrated assessment. After the diagnostic process, psychological techniques and interventions can be useful for pain management or, in case of surgical interventions, to enhance their outcomes.

## Economic benefits of an integrated assessment of pain and treatment outcomes and the role of modern psychometric methods

The multidimensional evaluation of pain and its correlates is crucial during the entire course of the care. Starting from the initial assessment phase, the aim of the pain specialist is to gather detailed information on pain characteristics and to ascertain how these characteristics are intertwined with biomedical, psychosocial and behavioral factors (Dansie and Turk, 2013; Aloisi et al., 2016; Castelnuovo et al., 2016a,b; Tamburin et al., 2016). An integrated assessment of these aspects may have an intrinsic positive clinical effect (Pietilä Holmner et al., 2013). In addition, accurate and objective measures are important for making correct decisions and to lead to a cost-effective management of the following pain management intervention. Standardized measures are fundamental for detecting the presence of contraindication for specific pain management options (Daubs et al., 2010). In this context, psychometrics may provide the tools for a reliable, sensitive and valid assessment of pain and of the outcomes of the treatment. Some authors advocate for the spread integrated and computerized assessment methods which exploit the potential of the most modern statistical models for the construction of valid, specific and user-friendly questionnaires which can be linked to automated dynamic pain assessment systems (Chang, 2013; El Miedany, 2013; Slover et al., 2015). Item Response Theory models can be used to calibrate these tools to assess the person's traits in a reliable and valid manner with the lowest possible amount of item, greatly reducing the administration time. These methods permit to evaluate the relevant aspects ofthe patient's experience and to easily store and access the acquired information throughout the different phases of the treatment and in the follow-up period. Models based on these principles have been specifically developed for musculoskeletal pain conditions with the aim to reduce costs and first proofs of their cost-effectiveness have been found (Wells et al., 2013; El Miedany et al., 2016).

## Economic utility of the assessment of the psychological variables associated with the treatment outcomes in the surgical management of pain

Surgery can be an option to relieve pain in rheumatoid arthritis, osteoarthritis and back conditions (Boonen and Severens, 2011; Gore et al., 2012; Xie et al., 2016). A large number of psychological aspects related to pain, such as anxiety, depression, cognitions, expectations and personality traits can be considered as strong predictors of the outcomes of these interventions (Schade et al., 1999; Trief et al., 2000; DeBerard et al., 2003; Kohlboeck et al., 2004; den Boer et al., 2006; Abbott et al., 2011; Judge et al., 2012; Block et al., 2013; Akins et al., 2015; Anderson et al., 2015; Kunutsor et al., 2016; Alattas et al., 2017; Lindberg et al., 2017; Mancuso et al., 2017). Each of these factors seems to differently affect the various outcomes of the treatment, leading to a boost of the direct and indirect costs of the care. Omitting to consider the psychosocial aspects which can interfere with the surgical intervention may lead to a worst patient experience in terms of pain intensity and quality of life, to a failure to return to work, to an increase in opioid consumption or to repeat other ineffective, potentially harmful and costly treatments. In this contexts, the contribution of a psychologist can be essential. His role is not to decide whether an intervention should be implemented or discarded, but to help physicians to identify the patients at risk of poor outcomes and to suggest how the pain management strategies could be improved. Moreover, his work can be fundamental to prepare the patient for the surgical intervention, e.g., assessing unrealistic expectations or providing education, and to guide him in the post-operative period with the aim to foster his motivation, to facilitate his discharge, and to prevent the conditions which may cause a relapse of the symptoms and a readmission to the hospital (Childs et al., 2014; Louw et al., 2014).

## The economic utility of clinical psychology for pain treatment

Several psychological treatment options have been proven to be cost-effective and are available for the clinical management of pain both in traditional and in new technology-based scenarios (Kröner-Herwig, 2009; Trompetter et al., 2014, 2015, 2016; Veehof et al., 2016). In a recent meta-analysis, Pike et al. (2016) found that psychological interventions are successful in reducing the use of healthcare services by the patients. This finding extends the evidence for a positive effect of psychological interventions on pain intensity, pain disability and the quality of life of the treated subjects (Hoffman et al., 2007; Williams et al., 2012; Veehof et al., 2016).

Comprehensive pain programs administered by multidisciplinary teams which include the contribution of a psychologist or which use psychological techniques are associated with a substantial reduction in both the direct and indirect costs of the disease, with a cost saving which is estimated between 8,500$ to 13,000$ per patient per year (Gatchel et al., 2003; Gatchel and Okifuji, 2006). All the components of these programs are fundamental for a cost-effective care of the disease and “carving out” some of them may impair a satisfying recovery to the premorbid productivity levels, leading to an increase in the future use of the healthcare resources (Gatchel and Okifuji, 2006; Gatchel and Mayer, 2008). Moreover, these programs may be enhanced providing intensive psychological therapies for the management of pain. The research is increasingly showing that these interventions are highly effective and lead to considerable cost savings. A group treatment for musculoskeletal pain sufferers based on cognitive behavioral principles resulted in additional 0.0325 Quality Adjusted Life Years (QALY) with respect of the control condition, with an incremental cost per QALY of £5,786 (Taylor et al., 2016). Various RCTs evaluated the cost-effectiveness of group cognitive behavioral approaches for chronic low back pain, with estimates of additional cost per QALY ranging from £1,786 to $7,197 (Linton and Nordin, 2006; Lamb et al., 2010; Norton et al., 2015). An integrated care program for sick-listed back pain patients based on a workplace intervention and graded activity was found to provide work-related economic savings in the amount of £5744 (Lambeek et al., 2010), but graded activity was found to be less cost-effective than exposure *in vivo* in another trial (Goossens et al., 2015). Non-significant effects were found for a CBT program added to inpatient rehabilitation for chronic low back pain (Schweikert et al., 2006). With regards to the other syndromes, a telephone-delivered CBT for chronic widespread pain sufferers provided a 0.097 additional QALY with respect to a program of tailored exercise, with an incremental cost per QALY of £5917 (Beasley et al., 2015), an internet-delivered Acceptance and Commitment Therapy program for fibromyalgia patients provided cost savings which exceeded the costs of the treatment 2 months after its conclusion (Ljotsson et al., 2014) and a psychoeducational intervention for the same syndrome resulted in 0.12 additional QALY with respect to control (Luciano et al., 2013). Although a systematic evaluation of the cost-effectiveness of all the available programs is beyond the scope of this article, it is established that the costs of various psychological treatments are rapidly overtaken by direct and indirect savings. However, clinical psychologists are not required to indiscriminately implement their therapies. On the contrary, their role is to help the pain management team to identify the characteristics of the patient and to tailor their techniques accordingly. The importance of tailoring the interventions has been long advocated in the literature and some evidence of the benefit of such an approach the have been provided (Turk, 1990; Turk et al., 1996, 1998). In addition, in the clinical practice, the psychologist and the multidisciplinary pain team usually face very complex conditions accompanied by physical or mental comorbidities, which may prevent the use of standardized treatments. The future of the clinical psychology and of the biopsychosocial approach in the field of pain management seems therefore to reside in the possibility to deliver integrated interventions which are personalized in order to be more effective and, at the same time, less expensive (Castelnuovo, 2010a,b; Castelnuovo et al., 2016c).

## Author contributions

All authors listed have made a substantial, direct and intellectual contribution to the work, and approved it for publication.

### Conflict of interest statement

The authors declare that the research was conducted in the absence of any commercial or financial relationships that could be construed as a potential conflict of interest.
